# Exercise for older people with heart failure and frailty: should we even try?

**DOI:** 10.1093/eschf/xvag265

**Published:** 2026-10-21

**Authors:** Sunanthiny Krishnan, Amye C N Goddard, Matthew Annals, Iain B Squire, Shirley Sze

**Affiliations:** Department of Cardiovascular Sciences, University of Leicester and NIHR Leicester Biomedical Research Centre, Glenfield Hospital, Leicester LE3 9QP, UK; Cardiac and Pulmonary Rehabilitation, Glenfield Hospital, Leicester LE3 9QP, UK; Centre for Exercise & Rehabilitation Science, NIHR Leicester Biomedical Research Centre-Respiratory, Glenfield Hospital, Leicester LE3 9QP, UK; Cardiac and Pulmonary Rehabilitation, Glenfield Hospital, Leicester LE3 9QP, UK; Centre for Exercise & Rehabilitation Science, NIHR Leicester Biomedical Research Centre-Respiratory, Glenfield Hospital, Leicester LE3 9QP, UK; Department of Cardiovascular Sciences, University of Leicester and NIHR Leicester Biomedical Research Centre, Glenfield Hospital, Leicester LE3 9QP, UK; Department of Cardiovascular Sciences, University of Leicester and NIHR Leicester Biomedical Research Centre, Glenfield Hospital, Leicester LE3 9QP, UK; Department of Population Health Sciences, University of Leicester, Leicester LE1 7RH, UK; Department of Cardiology, Glenfield Hospital, Leicester LE3 9QP, UK

**Keywords:** Heart failure, Frailty, Older people, Exercise

## Abstract

The benefits of exercise in older patients with heart failure (HF) and frailty are twofold: (i) improvement in cardiopulmonary capacity, thus reducing HF-related sequelae; (ii) attenuation of decline in physical function, thus improving frailty status. Despite the clear benefits, exercise training (ET) remains underutilized as a treatment modality in this patient population. Guidelines on the specific type of exercise intervention best suited for this population are still lacking. In this review, we discuss (i) pathophysiology underlying exercise intolerance in patients with HF; (ii) impact of frailty on HF and exercise capacity; (iii) benefits and safety of ET; (iv) recommended ET programme for this population; (v) mode of delivery; and (vi) potential barriers in the uptake of ET and strategies to overcome them. An effective ET intervention in this population warrants individualization to patient’s physical capacity, while addressing possible barriers to uptake so as to ensure sustained participation in the programme.

## Graphical Abstract

**Graphical Abstract:**
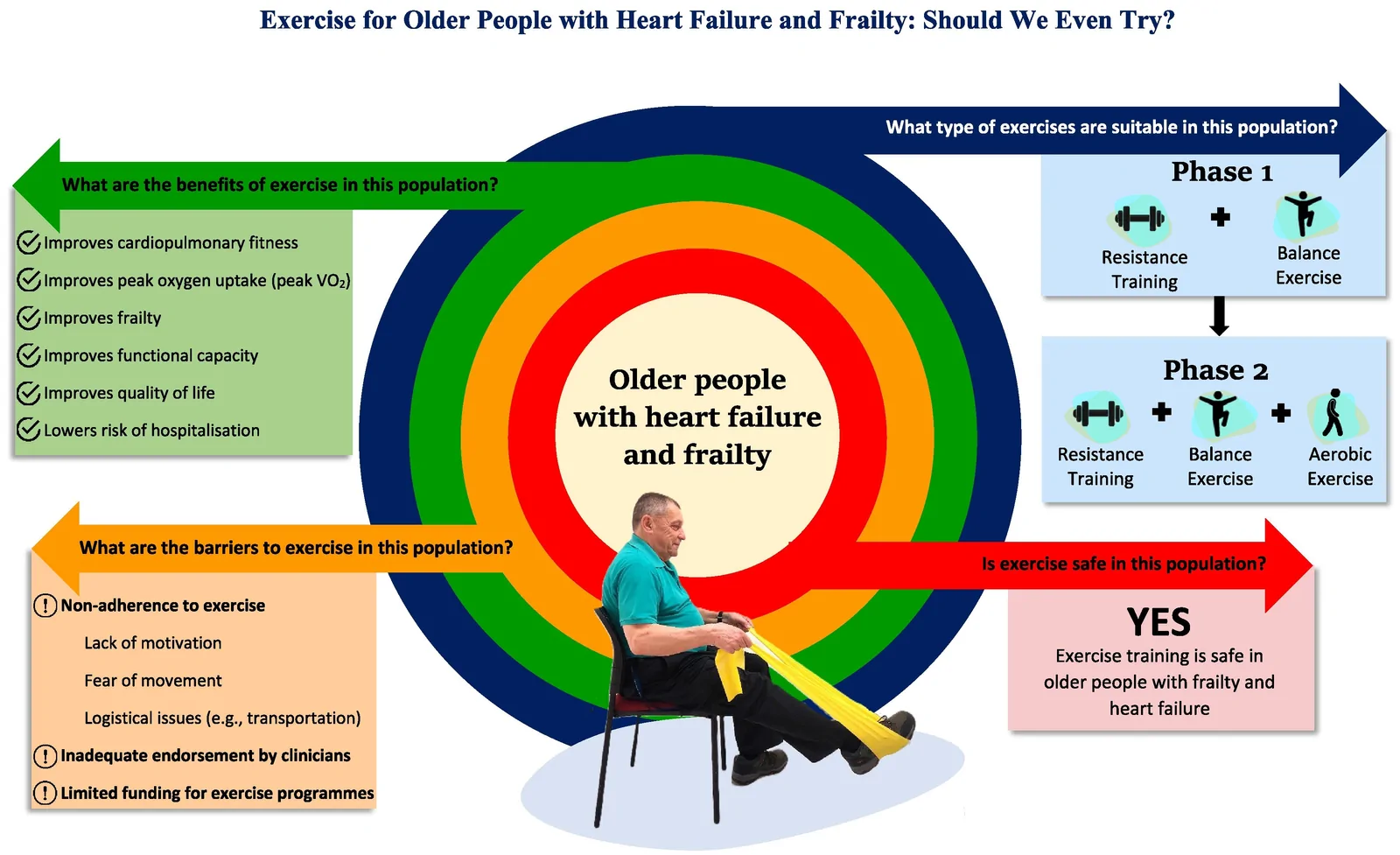

## Introduction

Benefits of exercise in patients with heart failure (HF) have been extensively documented in the literature. A most recent meta-analysis has shown that exercise-based cardiac rehabilitation not only improves health-related quality of life, but also reduces HF-related hospitalization and all-cause hospital admissions.^1^ Despite the robust evidence, exercise training (ET) is still largely underutilized in this cohort, particularly in older patients with frailty. Real-world data over the last 10 years indicate that only 25% of eligible patients with HF are referred to cardiac rehabilitation programmes. Older people, especially those with a high burden of comorbidities, are less likely to be referred.^2^

The low utilization of ET in this population is a cumulative effect of many factors, including poor integration of ET into current HF care pathways,^3^ limited resources,^3^ as well as poor patient motivation and adherence.^4–6^ Provider-related factors particularly low referral and inadequate physician endorsement, also play a crucial role.^7–9^ Concerns around safety and potential absolute benefit preclude the implementation of ET as a potent therapeutic intervention in this vulnerable group of patients.^3,5,8,9^ Furthermore, there is currently a lack of consensus on the ideal format of ET for patients with frailty and HF.

In this paper, we review the evidence on ET in HF and frailty and build a case for implementing ET in older people living with HF and frailty, with a specific focus on (i) pathophysiology underlying exercise intolerance in patients with HF; (ii) impact of frailty on exercise intolerance; (iii) benefits and safety of ET in HF and frailty; (iv) recommended ET programme for this population; (v) mode of delivery; and (iv) barriers in the uptake of ET and strategies to overcome them.

## Understanding the mechanism

Exercise intolerance due to fatigue and dyspnoea is a hallmark symptom of HF. The pathophysiology of impaired exercise capacity in patients with HF stems from a complex interplay between the cardiovascular, pulmonary, and musculoskeletal systems.^10,11^ In HF, the left ventricle (LV) loses its ability to accommodate the increased venous return during exercise, leading to a reduction in stroke volume.^12–15^ Coupled with lower maximal heart rate secondary to chronotropic incompetence, global cardiac output is attenuated and is, essentially, unable to meet the metabolic demands of the skeletal muscles, leading to exercise intolerance.^10,12–15^

As the working skeletal muscle switches to anaerobic metabolism in the face of reduced cardiac oxygen delivery,^15^ the ergoreceptors, particularly the metaboreceptors, are stimulated, mediating an ergoreflex that includes activation of sympathetic tone, vasoconstriction of non-exercising muscle vasculature as well as increased pulmonary ventilation.^16,17^ Ergoreflex hyperactivation seen in patients with HF contributes to an exaggerated increase in the ventilatory response to exercise, leading to respiratory muscle exhaustion, a major driver of exertional dyspnoea.^10,16,17^ Pulmonary venous congestion and increased ventilatory effort in response to high cardiac filling pressures are also strongly implicated in causing dyspnoea and reduced effort tolerance in patients with HF.^16,18^

Beyond the changes in metabolism, skeletal muscles of patients with HF have also been shown to undergo intrinsic adaptations that are contributory to decreased exercise capacity. Mitochondrial dysfunction, muscle atrophy with a reduction in muscle fibre size, as well as a shift in the fibre type, are closely linked with exercise intolerance.^10,19–23^ Muscle biopsy samples from patients with HF are characterized by decreases in Type I (slow-twitch) fibres and increases in Type II (fast glycolytic) fibres, the latter of which are easily fatigable.^20,23,24^

Despite these commonalities in the pathophysiology, their relative prominence varies between the HF phenotypes.^25^ In patients with reduced ejection fraction (HFrEF), reductions in stroke volume and cardiac output are more pronounced compared to those with preserved ejection fraction (HFpEF),^26,27^ a direct consequence of systolic dysfunction of the left ventricle. Conversely, extracardiac factors play a more pivotal role in HFpEF-mediated exercise intolerance. Evidence suggests that patients with HFpEF demonstrate significantly greater impairment in peripheral oxygen extraction,^27,28^ alongside worse muscle function and muscle atrophy compared to HFrEF.^29^ These reflect dominance of skeletal muscle and microvascular dysregulation in driving effort intolerance in this phenotype. Patients with HFpEF also experience disproportionately larger reduction in the left atrial reservoir function during exercise compared to HFrEF.^30^

## Dual burden of HF and frailty

In addition to HF-induced exercise limitations, the presence of frailty further compounds the physiological and functional burden in people living with both HF and frailty. A multidimensional, dynamic syndrome, frailty is characterized by a state of vulnerability due to reduced functional reserves of various physiological systems.^31^ With central features of weakness, decreased endurance and slowed performance, frailty is closely associated with HF, owing to overlapping pathophysiology, particularly chronic systemic inflammation, neurohormonal dysregulation and endocrine dysfunction.^32,33^ These processes aggravate changes in the structure and quality of skeletal muscle as a function of age, resulting in loss of muscle mass and strength.^34^ The decline in muscle mass places a compensatory demand on the remaining muscles to generate power to perform activities of daily living (ADL), leading to early fatigability.^35^

Frailty invariably leads to a decline in functional independence and quality of life of older patients with HF.^32,36–38^ Moreover, frailty in HF is associated with a poorer prognosis. A meta-analysis by Yang and colleagues reported that frailty increases the risk of mortality and incident hospitalization by about 1.5-fold within 2 years of follow-up.^39^ The cumulative incidence of all-cause mortality and recurrent HF hospitalization are incrementally higher in worsening frailty status, as assessed using the Clinical Frailty Scale (CFS).^40^

Whilst the prevalence of frailty is generally higher in the HFpEF population (due to a typically older demographic with a higher burden of comorbidities),^32^ the prognostic outcomes are consistent across the phenotypes. In HFpEF, greater severity of frailty (classified as CFS ≥4) is associated with increased hazard of all-cause mortality (adjusted HR 2.54, 95% CI 1.39–4.66, *P* = .003) and HF hospitalization (adjusted HR 1.55, 95% CI 1.03–2.32, *P* = .035) compared to lower CFS class.^41^ Likewise, in a pooled analysis of two HFrEF trials, i.e. PARADIGM-HF and ATMOSPHERE, frail patients (identified using a 42-item Frailty Index) were found to have increased risk of composite outcome of cardiovascular death/HF hospitalization (adjusted HR 1.24, 95% CI 1.14–1.35, *P* < .001), all-cause death (adjusted HR 1.26, 95% CI 1.14–1.39, *P* < .001) and recurrent all-cause hospitalizations (adjusted HR 1.24, 95% CI 1.16–1.33, *P* < .001). The risk estimates were significantly higher in frailer patients.^42^ HFrEF patients with a high burden of frailty are also less likely to receive optimal guideline-directed medical therapy.^43,44^ This is of relevance as evidence suggests mineralocorticoid receptor antagonists, a commonly underutilized therapy in HF, improves exercise capacity in this population.^45,46^

## Quantifying exercise intolerance

The degree of exercise intolerance in HF is often quantified in terms of peak oxygen uptake (peak VO_2_), measured during a cardiopulmonary exercise test (CPET). Defined as the highest rate of oxygen consumption attained during physical exertion, peak VO_2_ reflects the combined functional capacity of cardiovascular, pulmonary, and musculoskeletal systems.^47^ Patients with HF generally have low peak VO_2_, often below the threshold for functional independence, resulting in many ADLs requiring near or maximal effort.^48^ Presence of comorbidities such as atrial fibrillation,^49^ renal dysfunction,^50^ and chronic obstructive pulmonary disease^51^ further compromise their exercise tolerance. This is likely to be worse for patients living with physical frailty.

In a secondary analysis of HF-ACTION, the largest trial to date on ET in patients with HF, the mean peak VO_2_ of patients with frailty was 14.1 mL/kg/min^52^; the study population was exclusively of patients with HFrEF. A similar value is reported in HFrEF patients from the MECKI HF Italian registry (i.e. 14.2 mL/kg/min), with progressive reduction across older age categories.^53^ Among patients with HFpEF, the value reported is comparable at 14.9 ml/kg/min.^48^ For context, the average peak VO_2_ of a healthy older adult (aged ≥70 years without any cardiovascular, metabolic or mental illnesses) is about 20–25 mL/kg/min; values are typically lower in women.^54^

With reference to metabolic equivalents (METs) of physical activities in older adults,^55^  *Figure 1* illustrates the VO_2_ required for several common activities relative to the peak VO_2_ of a patient living with HF and frailty (VO_2_ = MET × 3.5 mL/kg/min^47^). It is worth noting that a task as simple as walking demands maximal physical exertion in this population. This underscores the need for a conservative approach in prescribing ET in these patients, especially during the initial phase, with careful consideration of the type and intensity of exercises.

**Figure 1:**
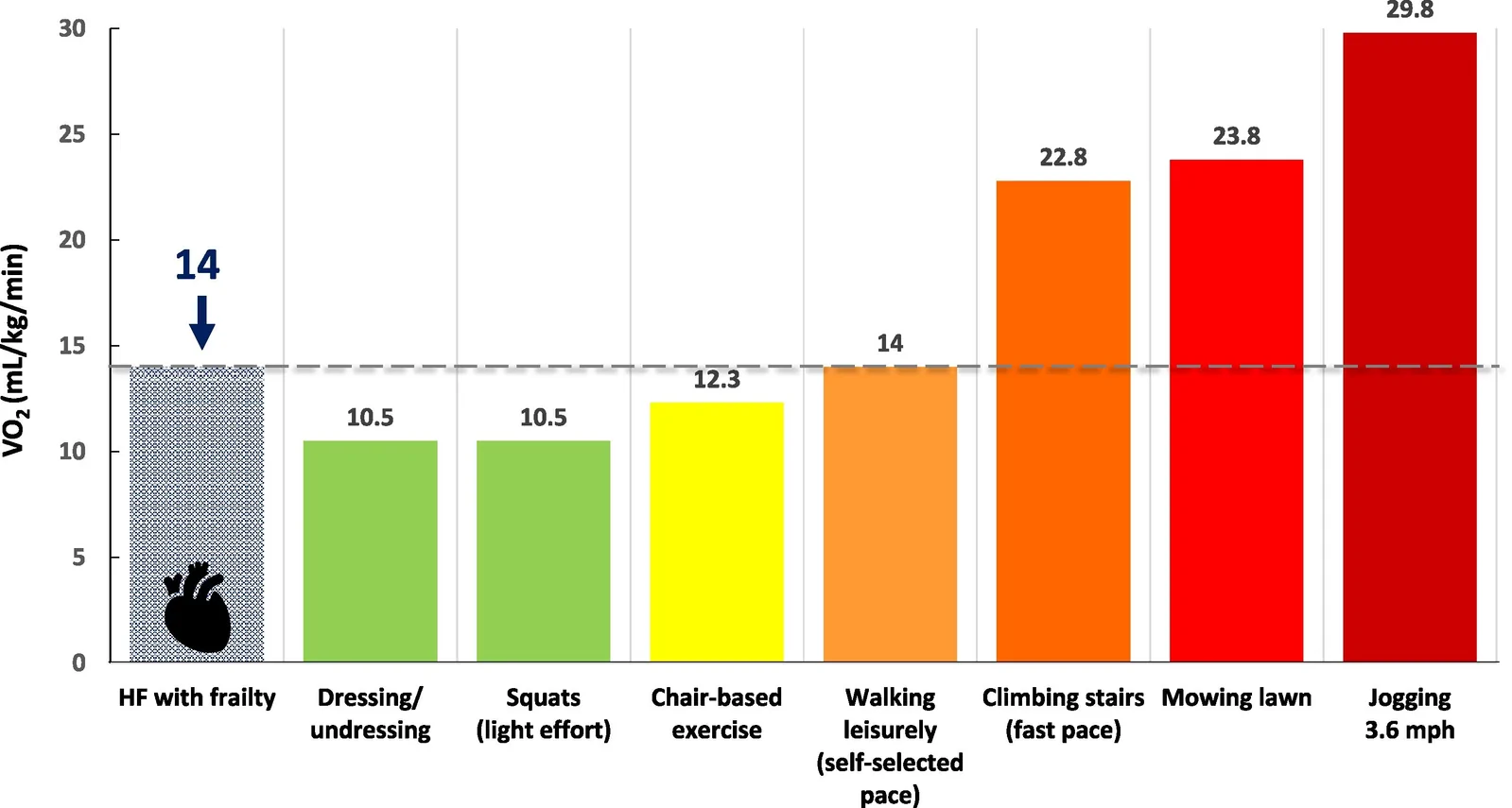
Oxygen uptake (VO_2_) required for common daily activities relative to the peak VO_2_ of a patient living with HF and frailty

## Benefits of exercise in HF and frailty

The benefits of ET in older patients with HF and frailty are two-fold: (i) improved cardiopulmonary fitness and (ii) attenuation of the decline in physical function, thus improving frailty status. From the cardiorespiratory perspective, ET has been shown to clinically improve exercise capacity.^56,57^ These benefits are mechanistically driven by improvements in cardiac output, anaerobic threshold, muscle mass and fibre composition as well as strength, attenuation of neurohormonal activation and improvement in endothelial function.^7,56–62^ Importantly, ET improves peak VO_2_ in patients with HF. A meta-analysis of 84 randomized controlled trials demonstrated that exercise increased peak VO_2_ by 2.98 mL/kg/min (95% CI 2.52–3.43, *P* < .001) compared to the non-exercise group.^63^ Gains in peak VO_2_ following ET are evident in both HF phenotypes.^26^

In the context of frailty, a wealth of published literature shows that ET is an effective treatment modality to improve functional capacity.^64–67^ A well-structured exercise programme increases gait speed, balance, mobility and muscular strength, translating to improved ability to perform ADLs,^65–69^ and has also been demonstrated to reduce risk of falls.^69–71^ In a secondary analysis of REHAB-HF, a landmark trial on physical rehabilitation among older patients with acute decompensated HF, patients with worse baseline frailty status had significantly greater improvement in physical function following the intervention, attesting to the added advantage of ET on patients with frailty.^72^ The benefits of ET on peak VO_2_ are also proven in patients with HF and frailty, with a similar quantum of increment as non-frail patients.^52^

Naturally, the therapeutic effects seen with ET translate to enhanced quality of life (QoL) in these patients. As evidenced by the latest Cochrane review (60 randomized trials; 8728 patients; predominantly HFrEF), participation in ET is associated with clinically significant improvement in health-related QoL.^1^ Baral and colleagues also affirmed similar improvements in HFpEF patients.^73^ Whilst the merits of exercise on the QoL of frail older adults remain to be solidified,^65,74^ there is strong evidence to support that improvement in physical functions is associated with better QoL in this population.^74^

In terms of clinical outcomes, participation in ET is associated with lower risk of hospitalization in patients with HF. Notably, ET is likely to reduce the risk of all-cause and HF-related hospitalization by 31% and 18%, respectively, at up to 12 months of follow-up.^1^ It may not confer substantial survival benefits in this population,^1^ although improvements in peak VO_2_ may alter mortality risks. Specifically, for every 6% increase in peak VO_2_ following ET, the risk of all-cause mortality is reported to be lowered by 7% and cardiovascular mortality or HF hospitalization lowered by 8%.^75^

Given the compelling evidence on functional status, QoL and hospitalization, ET is strongly recommended to all patients with HF by both North American^76^ and European^77^ cardiovascular guidelines (Class 1A Recommendation).

## Safety of exercises

Historically, the safety of ET among older adults with frailty has been a major concern owing to perceived predisposition to higher risk of adverse events. However, systematic reviews of published literature have demonstrated that ET is safe and effective in the frail population.^78,79^ Furthermore, the pivotal HF-ACTION trial showed that prescribed aerobic ET is not only safe but also resulted in a statistically significant reduction in all-cause hospitalisation, particularly in frail participants. Of note, 59% (1266 out of 2130) of the study participants were characterized as frail.^52^

It is crucial that an older person undergoes thorough assessment by a rehabilitation specialist, at the outset, to rule out the presence of any contraindications. *Table 1* illustrates the guidelines specified by leading cardiovascular societies on contraindications to ET. In the presence of a contraindication, rehabilitation specialists are urged to use their clinical judgment to modify the ET programme, as deemed appropriate, to suit the patient’s health condition, rather than categorically dismissing exercise as inappropriate.

**Table 1:** Contraindications to exercise training in cardiovascular population as outlined by key professional bodies

British Association for Cardiovascular Prevention and Rehabilitation (BACPR 2023) and Association of Chartered Physiotherapists in Cardiovascular Rehabilitation (ACPICR 2023) (*In line with American College of Sports Medicine 2021*^80^*)*	Heart Failure Association (HFA)/European Association for Cardiovascular Prevention and Rehabilitation (EACPR), 2011^81^	American Heart Association (AHA) and American Association of Cardiovascular and Pulmonary Rehabilitation (AACPR)^82^ *(Adopted by American College of Sports Medicine 2021*^80^*)*
**Contraindications**	**Absolute contraindications**	**Contraindications**
Unstable angina Uncontrolled hypertension (resting SBP >180 mmHg and/or resting DBP >110 mmHg) Orthostatic BP drop of >20 mmHg with symptoms Significant aortic stenosis (aortic valve area <1 cm^2^ Uncontrolled atrial or ventricular arrhythmias Uncontrolled sinus tachycardia (>120 bpm) Uncompensated HF Third-degree atrioventricular block without pacemaker Active pericarditis or myocarditis Recent embolism (pulmonary or systemic) Acute thrombophlebitis Aortic dissection Acute systemic illness or fever Uncontrolled diabetes mellitus Severe orthopaedic conditions that would prohibit exercise Untreated metabolic conditions, e.g., thyroiditis, hyper/hypokalaemia or hypovolaemia Severe psychological disorder	Early phase after acute coronary syndrome (up to 2 days) Untreated life-threatening cardiac arrhythmias Acute heart failure (during the initial period of haemodynamic instability) Uncontrolled hypertension Advanced atrioventricular block Acute myocarditis and pericarditis Symptomatic aortic stenosis Severe hypertrophic obstructive cardiomyopathy Acute systemic illness Intracardiac thrombus Progressive worsening of exercise tolerance or dyspnoea at rest over the previous 3–5 days Significant ischaemia during low-intensity exercise (<2 METs, <50 W) Uncontrolled diabetes Recent embolism Thrombophlebitis New-onset atrial fibrillation/atrial flutter	Unstable angina Uncontrolled hypertension (resting SBP >180 mmHg and/or resting DBP >110 mmHg) Orthostatic BP drop of >20 mmHg with symptoms Significant aortic stenosis (aortic valve area <1 cm^2^ Uncontrolled atrial or ventricular arrhythmias Uncontrolled sinus tachycardia (>120 bpm) Uncompensated HF Third-degree atrioventricular block without pacemaker Active pericarditis or myocarditis Recent embolism (pulmonary or systemic) Acute thrombophlebitis Aortic dissection Acute systemic illness or fever Uncontrolled diabetes mellitus Severe orthopaedic conditions that would prohibit exercise Untreated metabolic conditions, e.g., thyroiditis, hyper/hypokalaemia or hypovolaemia Severe psychological disorder
	**Increased risk for ET**	
	>1.8 kg increase in body mass over the previous 1–3 days Concurrent, continuous, or intermittent dobutamine therapy Decrease in systolic blood pressure with exercise NYHA functional class IV Complex ventricular arrhythmia at rest or appearing with exertion Supine resting heart rate >100 bpm Pre-existing co-morbidities limiting exercise tolerance	

**BP**, blood pressure; **SBP**, systolic blood pressure; **DBP**, diastolic blood pressure; **METs**, metabolic equivalents; **W**, watt.

Use of cardiac implantable electronic devices does not preclude patients from engaging in ET. A systematic review of 16 studies (with over 2500 patients), has established that exercise intervention in patients with implantable cardioverter-defibrillators (ICDs) and cardiac resynchronization therapy with defibrillator (CRT-D) is safe and effective.^83^ It is postulated that ET may even be protective in this population by reducing ICD shock burden (both appropriate and inappropriate), possibly through regulation of autonomic tone resulting in reductions in ventricular arrhythmias, sinus tachycardia and supraventricular tachycardias. Meta-analysis shows that the rate of ICD discharges in patients receiving ET is lower compared to those who do not engage in any exercise programme (OR 0.7, 95% CI 0.53–0.92, *P* = .013).^83^

In most cases, it is the fear of shocks during exercise that prevents patients from participating in ET. This, however, can be mitigated with appropriate device programming and effective patient monitoring. Consensus documents mandate a symptom-limited CPET in patients with ICD prior to commencement of an exercise programme.^81,84^ This is to evaluate patients’ chronotropic response to exercise, presence of exercise-induced arrhythmias, and to test device settings, particularly the risk of reaching an exercise heart rate (HR) in the ICD intervention zone. In order to prevent inappropriate shocks, it is recommended that exercise level for patients with ICD be limited to a maximal HR of 10–20 bpm below the programmed intervention zone for the device.^81,84^ In addition, wearables such as smartwatches or fitness trackers could be used to monitor HR during exercise to reduce the risk of inappropriate shocks.

## Type of exercise

Whilst the evidence is clear on the positive effects of exercise in older patients with HF and frailty, the specific type of exercise intervention best suited for this population remains less clear.^65–67^ The task force of the International Conference of Frailty and Sarcopenia Research (ICFSR) recommends a multi-component physical activity programme for all older adults with frailty that combines resistance training with aerobic and balance training for a holistic effect on muscle strength, coordination and cardiopulmonary fitness.^85^ The ESC Guideline on Sports Cardiology and Exercise has a similar recommendation for older patients with cardiovascular disease, with a somewhat greater emphasis on aerobic exercises for patients with HF,^86^ supported by data from a substantial number of studies employing aerobic/endurance exercise interventions within the HF space.^1,86^

### Resistance exercise

There is compelling evidence to support attenuation by resistance training (RT) of the effects of ageing on neuromuscular function and functional capacity, and thus attenuating physical frailty.^87–89^ Resistance training improves muscle strength, muscle mass, gait speed, and dynamic balance^88^ without excessive stress on the heart.^81^ Furthermore, it is also effective in improving peak VO_2_ in patients with HF without adversely affecting cardiac remodelling, contrary to historical concerns.^90^

Over the last decade, eccentric-focused RT has gained considerable traction as a more favourable exercise intervention to improve muscle strength and physical functioning in older adults. Conventional RT emphasizes more on concentric muscle contraction in which the target muscle fibres are shortened as force is generated. In contrast, during eccentric contraction, the muscles are intentionally lengthened by applying greater resistive force relative to the contractile force of the muscles (*Figure 2*).

**Figure 2:**
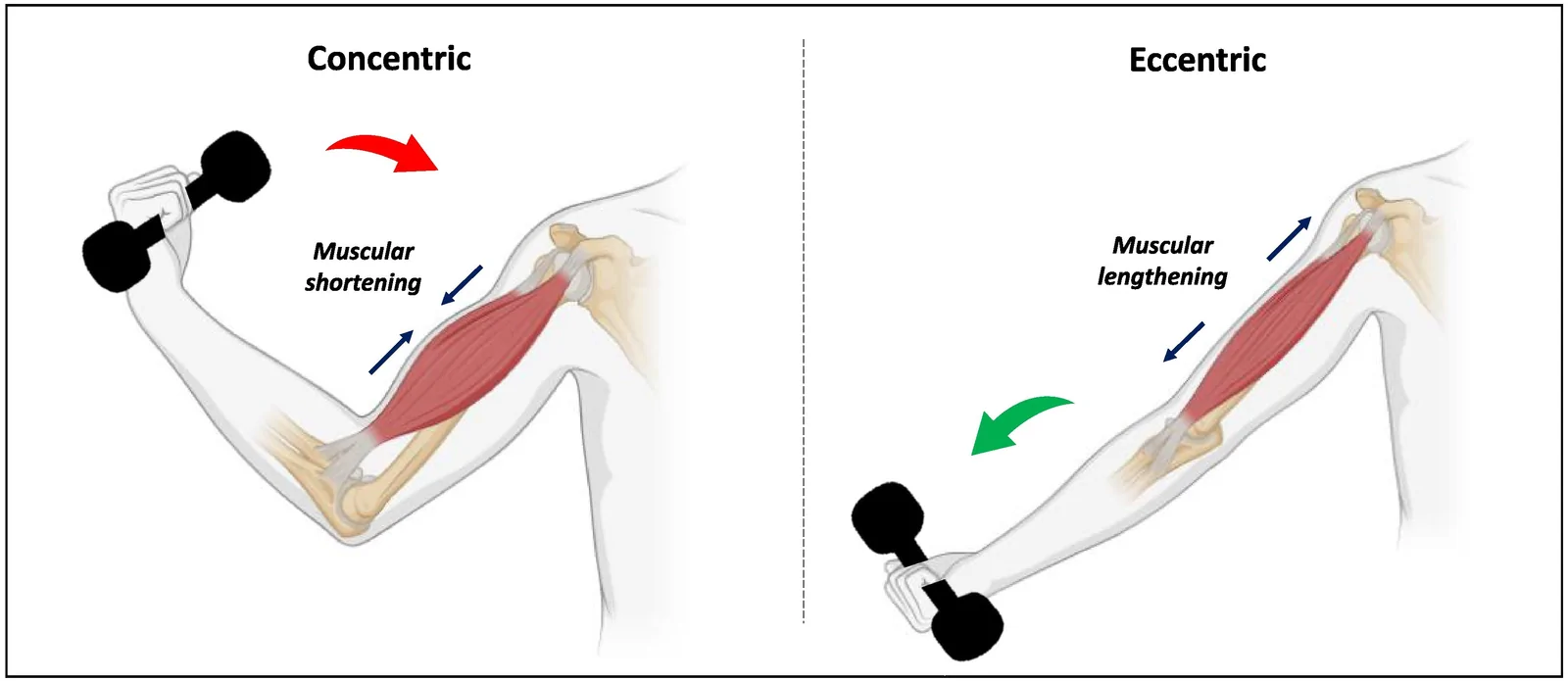
Muscle contraction during resistance training

In terms of muscle energetics, the energy required (i.e. energy cost) for production of force in muscles is greater during muscle shortening compared to lengthening, rendering eccentric contraction more cost-efficient.^91,92^ Mounting evidence demonstrates that emphasizing eccentric muscle contraction during RT is more effective in improving lower limb strength, mobility and postural stability in older people compared with the traditional/concentric training.^91,93,94^

The intramuscular and metabolic mechanisms unique to eccentric training offer several advantages to older people, commensurate with the skeletal muscle changes typical of ageing. While it is acknowledged that there is a progressive reduction in contractile capacity in ageing muscles, there is relative preservation of eccentric strength compared to concentric or isometric torque.^95^ This makes eccentric training interventions particularly attractive in older people with reduced levels of strength.^91,95^ Moreover, eccentric muscle contraction utilizes low energy to generate high levels of force.^91,92^ This low metabolic cost of eccentric exercise translates to reduced cardiorespiratory burden, making it a more suitable type of RT for patients with limited exercise tolerance, such as those with HF.^95,96^

Traditional RT can be modified to be eccentric-focused by lengthening the eccentric phase of muscle contraction. For instance, during bicep curls, the arms are lowered at a controlled pace, slower than the time taken to curl up. During sit-to-stand, extending the time to lower oneself down will shift the focus to eccentric muscle activation.^93^ For more exclusive eccentric training delivery, specialized equipment such as the Eccentron^TM^ can also be considered.

Resistance training should involve all major muscle groups (shoulders, arms, chest, abdomen, back, hips, and legs) with progressive overload to promote muscle adaptation and thus build strength.^97^ Progression can be achieved by increasing the number of sets, repetitions, or intensity of the exercise, guided by the one-repetition maximum (1RM), an objective measurement of muscle strength. In a resource-limited setting, subjective assessment using rating of perceived exertion (RPE) can be used as an alternative to gauge submaximal workload and inform exercise progression.^86,89^ The Borg RPE is one such tool that is widely used in practice to assess an individual’s perception of effort on a scale of 6 (no exertion at all) to 20 (maximal exertion).^98^

It is important that progression of RT is established strictly according to the individual patient’s tolerance to the intensity/volume of exercise. In older patients, it is recommended that a slower pace of progression is adopted, starting with low intensity to allow time for familiarization and adaptation. Too much, too soon carries a high risk of injury, particularly in frail patients whose muscles are typically unconditioned to rigorous exercise due to prolonged disuse, and may also be counterproductive in sustaining motivation to ET in the long run. Gradual progression at 2–4-week intervals has been previously reported.^93^

### Balance exercises

Reinforcement of balance and co-ordination is an integral component of ET to reduce the risk of falls in older people with frailty.^99,100^ Balance training based on principles of Tai-Chi^101^ is shown to reduce risk of falls and fear of falling. Whilst yoga improves balance and reduces fear of falling,^102,103^ robust evidence is still lacking on the effect of yoga on falls prevention in older adults.^104^ Similarly, pilates-based interventions improve postural stability, gait and fear of falling in older people, although high-quality evidence with regard to its effectiveness in reducing falls is still scant.^105^ The Otago Exercise Programme (OEP) is another extensively adopted exercise modality that arguably has the most consistent evidence to date in falls prevention amongst older adults with frailty.^99^

The OEP is a structured falls prevention programme that has shown an overall falls reduction and fall-related injury reduction by 35% in older adults.^106–108^ Notably, the programme is most effective in older people with a history of previous of falls, preventing 54 falls per 100 person-years in those ≥80 years old and 44 falls per 100 person-years in those aged ≥65.^108^ The programme has 17 simple exercises designed to improve balance and lower limb strength. It is a low-cost exercise modality that can be conducted either as a group session or individualized for home-based training.

The effect of OEP on older people with frailty has also been studied. A recent meta-analysis that included five studies showed that OEP is effective in reducing frailty, alongside improving physical balance and handgrip strength in older people with frailty or pre-frailty.^109^ A study on 18 older adults with cognitive impairment has shown that group-based OEP improved frailty (in terms of Fried Phenotype score) following a 12-week intervention, 3 sessions/week.^110^ This demonstrates OEP is safe and feasible even in the most vulnerable population.

### Aerobic exercise

Various forms of exercise, such as walking, jogging, cycling, water aerobics, swimming and dancing, have been proposed to improve aerobic capacity in older adults.^80,86,111^ These exercises exert beneficial effects on the cardiopulmonary system, predominantly by way of increasing the peak VO_2_.^7,63,112^ The general recommendation for adults aged ≥65 years is to participate in 150 min of moderate-intensity aerobic exercise a week.^80,86^ For older patients with HF, this might be an ambitious goal to achieve all at once given their low cardiorespiratory fitness and typically sedentary lifestyle. It is therefore important that, in the case of older adults with HF and frailty, a more conservative approach is adopted with a gradual increment in volume over time. The aim should be to achieve an exercise intensity that optimizes cardiovascular fitness gain whilst retaining tolerability and acceptability, the latter of which is pivotal for long-term adherence to the ET.

Essentially, an exercise that is not too metabolically demanding will be favourable in this population so that it can be continued for a sufficient period of time to stimulate aerobic adaptation. Of the various exercise modes recommended, walking is probably the most feasible form of aerobic exercise for older people, particularly for those with severe functional impairment. It is simple, low-impact, inexpensive, and freely accessible without the need for professional supervision. More importantly, it is functionally the most relevant exercise for their ADLs. Moreover, physical deficits secondary to frailty could pose a challenge in effectively engaging in other traditional endurance exercises. The benefits of walking as a form of physical activity are multifaceted and well-recognized. In older adults, walking on a regular basis improves physical performance, fitness and prevents incidence of physical disability.^113,114^

In terms of frailty, walking alone may be insufficient but walking in combination with resistance exercises harbour potential benefit in lowering the risk of physical frailty.^115^ In a group of older men with HF, Volterrani and colleagues demonstrated that concurrent aerobic and resistance training yielded significantly greater muscular strength and functional capacity compared to aerobic training alone,^116^ reinforcing the relevance of multimodal exercise interventions for targeting physical frailty in HF.

*Table 2* summarizes recommendations for the various types of exercises in the HF population and in older adults. It is important to recognize that these population-based guidelines are often unfeasible in patients living with frailty. Instead of applying them prescriptively, clinicians are advised to prioritize practicality and adapt the protocols to suit patients’ functional reserve. The aphorism of geriatric medicine, ‘start low, go slow’ is highly applicable in this context. *Figure 3* illustrates a practical guide to initial exercise prescription in older people with HF and frailty.

**Figure 3:**
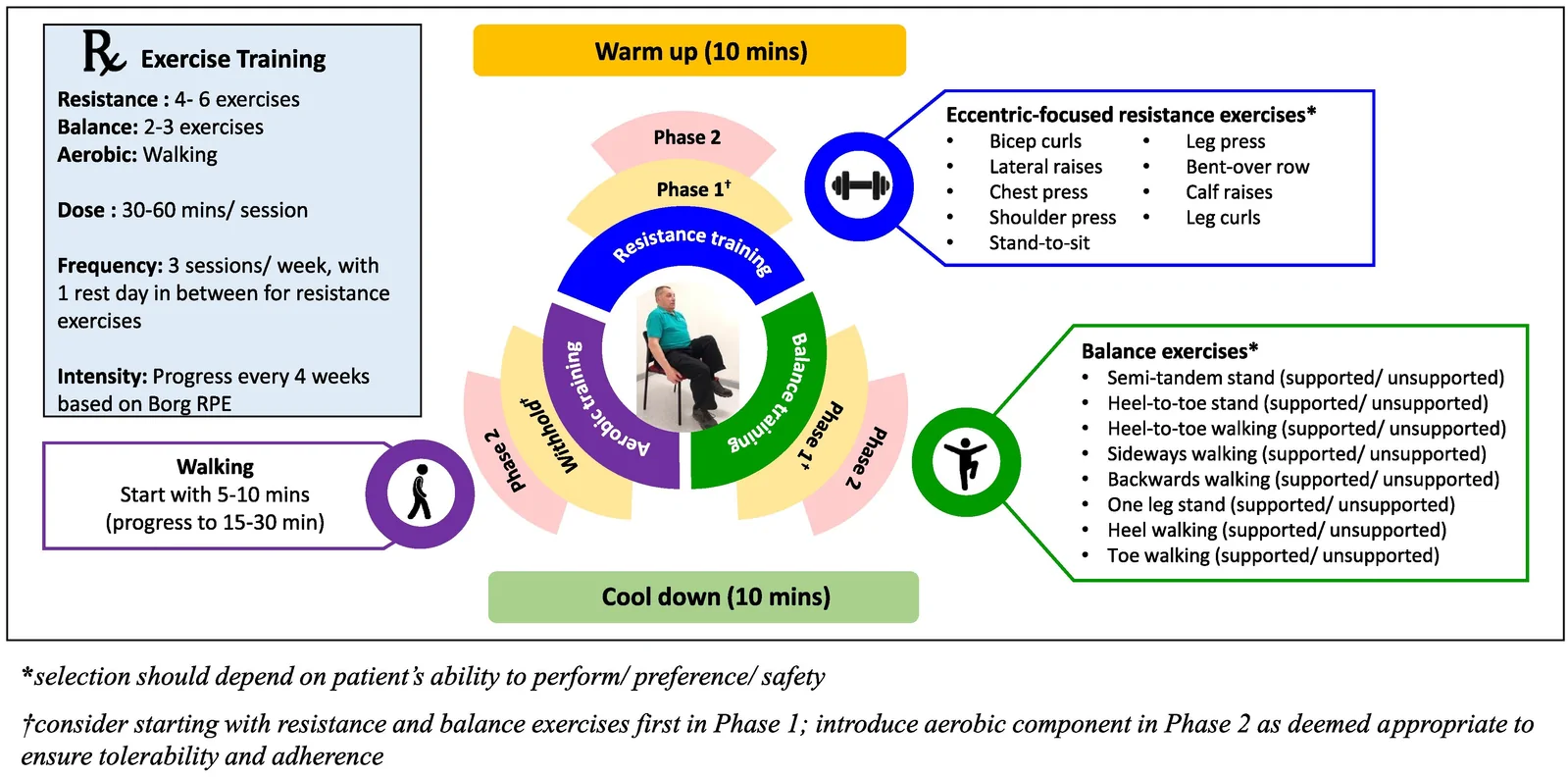
Proposed exercise prescription in older adults with HF and frailty

**Table 2:** Exercise recommendations for HF population and older adults >65years

Exercise prescription	Heart failure population^80,86^	Older adults^80,86,89,111^
**Resistance exercise**		
**Frequency**	1–2 days/week (non-consecutive days)^80^ 2–3 days/week^86^	At least 2 days/week^86^ 2–3 days/week, per muscle group (non-consecutive days)^89,111^
**Intensity**	40% of 1RM for upper body; 50% for lower body^80^ Borg RPE <15 or 40–60% of 1RM^86^	Start at 20–30% of 1RM and progress to 70–85% of 1RM^89^ Start at 30–40% of 1RM and progress to 70–80% 1 RM or 15–18 on Borg RPE^111^
**Type of exercises**	8–10 different upper and lower body exercises^80^	8–10 types^86,89,111^
**Sets**	2 sets^80^ At least 1 set^86^	1 set for beginners and older adults with frailty progressing to multiple sets^89^ 1–3 sets^111^
**Repetitions**	10–15 reps^80,86^	8–12 reps with variation for muscular strength for healthy older adults^89,111^ 10–15 reps at a lower relative resistance for beginners^86,89^
**Progression**	A progressively increasing training regimen should be prescribed (at least every 3–6 months) to adjust the duration and level of exercise to the reached level of tolerance	Progress slowly
**Balance exercise**		
**Frequency**	***No specific recommendation***	At least 2 days/week^86^ 1–7 days/week^111^
**Sets**		1–2 sets^111^
**Types of exercises**		4–10 different exercises emphasizing static and dynamic postures^111^
**Progression**		Progressive difficulty by narrowing the base of support/decreasing sensory input (e.g. closing eyes)/dual-tasking (e.g. adding a cognitive task while tandem walking)^111^
**Aerobic exercise**		
**Frequency**	3–5 days/week^80,86^	3–7 days^111^ Moderate: 5 days/week or vigorous: 3 days/week^80,86^
**Intensity**	Borg RPE 11–14^80^ 40–80% peak VO_2_^86^	Moderate: Borg RPE 11–13; Vigorous: Borg RPE 14–16^80,86^ Borg RPE 12–14^89,111^
**Time**	30–60 min/day^80^ 20–60 min/day^86^	20- 60 min/day^111^ ≥150 min/week^80,86^
**Type of exercises**	Treadmill, free walking, stationary cycling^80^	Step-up, stair climbing, recumbent cycling, walking^80,86,111^
**Progression**	***No specific recommendation***	Start at 5–10 min and progress to 15–30 min^89,111^

***Adapted from*:** American College of Sports Medicine^80^; Pelliccia et al.^86^; Fragala et al.^89^ & Izquierdo et al.^111^

**RPE**, ratings of perceived exertion; **1RM**, one-repetition maximum.

## Mode of delivery

Delivery of ET for patients with HF may vary across health systems according to availability of infrastructure.^3,117,118^ In the UK, it is largely delivered in an outpatient setting as a component of cardiac rehabilitation programme following hospital discharge when patients are medically stable.^118,119^ Depending on suitability and accessibility, patients may choose to attend sessions at centres based in hospitals or in the community upon referral by their physician. In some cases, home-based programmes are offered, supervised by relevant healthcare professionals.^118,119^

Inpatient or residential rehabilitation programmes, more common in other European countries,^118^ are particularly suitable for high-risk patients requiring closer monitoring such as those with advanced HF. This includes patients with NYHA class III/IV symptoms, needing continuous/intermittent drug infusion and/or after device implant.^117^ It is also a preferred mode of delivery for older people with frailty and for those unable to attend outpatient progammes.^117^

## Barriers to exercise

The benefits of ET are strongly dependent on the level of compliance to the programme.^120^ Arguably, older people with HF and frailty are amongst the most challenging patients in whom to initiate and maintain an ET programme. Adherence rates of ET in the HF cohort are generally low.^4^ In the HF-ACTION study, only 42% of participants were fully adherent to the supervised ET within the first 3 months of the programme. Subsequently, the adherence rate declined.^121^ The REHAB-HF trial reported a higher adherence rate of 67%.^120^ It should, however, be noted that older patients who were ADL-dependent or unable to walk at least 4 metres were excluded from the study.^122^ As such, the possibility of a lower adherence rate among patients with poorer physical frailty cannot be ruled out.

Intrinsic factors such as lack of motivation, poor self-efficacy and fear of movement (due to risk of injury or worsening HF symptoms) are frequently cited as barriers to exercise in this patient population.^4–6,123^ Externally, transport issues and lack of family support further deter patients from engaging fully in ET.^123^ Navigating these complexities may require considerable time and effort on the part of the treating physician and rehabilitation specialist, but one that is necessary to ensure these patients are not deprived of the benefits of ET. A systematic approach starting with raising awareness about the benefits of ET and eliciting perceived barriers will afford better adoption of ET among these patients. In order to increase motivation, the self-determination theory posits that implementation of strategies that support one’s autonomy, competence and relatedness will yield favourable results.^124^ In other words, a programme that is tailored to align with patients’ capacity, preferences and personal goals will enhance their motivation and lasting uptake of ET.

At provider-level, inadequate endorsement and thus low referrals to ET programmes by clinicians remain as important determinants of underutilization of ET in this patient population. This is largely the result of limited knowledge on the benefits and safety of exercise coupled with the general lack of awareness of guidelines on ET prescription for HF.^3,7–9^ In many settings, a clear pathway for referral to ET is lacking.^3^

Deficits at system-level such as shortages in funding, human resources, and infrastructure also continue to challenge efforts in implementing ET programmes in this population. Findings from the ExTraHF survey, a study on ET that included 170 cardiac centres around Europe, indicated that as many as 67 centres did not have any form of ET for patients with HF; 25% was due to lack of resources, the leading cause of non-implementation.^3^ This clearly highlights the need for a concerted effort in structured dissemination of knowledge to clinicians and stakeholders alike, to drive ET to the forefront of HF management, supported by strategic funding allocations.

## Practical considerations

An effective ET programme is one that is tailored to the individual patient, built to complement their present physical capacity. For instance, in patients with severe effort intolerance due to poor cardiac function, it is likely to be futile to push for aerobic exercise at the start of an exercise programme. Instead, starting with low-intensity, eccentric-focussed resistance training will help improve their peak VO_2_ without excessive stress on the heart. Over time, this will build sufficient cardiorespiratory strength to then slowly incorporate aerobic exercise.

Similarly, in frail patients with limited mobility, building strength and balance of the lower limbs should take precedence so that they can effectively partake in aerobic exercises with minimal risk of fall/fear of falling. For those who are very deconditioned or have a chronic sedentary lifestyle, ‘exercise snacks’ (i.e. short, frequent bouts of physical activity dispersed throughout the day) would be a more viable approach to introduce and integrate physical activity into their daily routine.^125^

Where inpatient ET programmes are unavailable, home-based, seated exercises might be an option for the very frail older adults with limited mobility and/or transportation to get to exercise centres. Notably, home-based ET programmes are equally effective as traditional, centre-based programmes in improving QoL and reducing hospitalisation.^1^ Alternatively, a hybrid mode (i.e. combination of home- and centre-based) or digitally-supported delivery could be explored to facilitate uptake of ET in this population.

Both frailty and HF are clinically dynamic syndromes with oscillating symptom severity. As such, understanding of patients’ baseline symptoms (particularly peripheral oedema, weight and fatigue) is prudent to effectively gauge their response to exercise as these symptoms have a negative impact on their exercise tolerance. A flexible exercise prescription that accommodates fluctuations in patients’ symptoms and their concomitant conditions is sensible to ensure sustained engagement in the programme.

## Conclusion

Older adults with frailty represent a significant proportion of patients living with HF. They are invariably trapped in a vicious cycle of physical deconditioning due to poor cardiopulmonary capacity. Reduced effort tolerance from HF causes a decline in physical function. This accelerates cardiac impairment, which in turn leads to further worsening of effort tolerance. In frail patients, the deterioration is more rapid due to deficits in their functional reserves. Based on current literature, ET is plausibly the most effective modality to break this cycle. Exercise training improves cardiopulmonary fitness in parallel with physical capacity in older people living with HF and frailty. By negating the decline in physical functions, ET is integral in reversing frailty. Importantly, ET is safe in older people with frailty. A well-structured ET is one that is individualized to patient’s physical status, incorporating progressive resistance training, balance, and aerobic exercises. Whilst adherence to ET could be challenging in this population, understanding their preferences and personal goals will likely ensure long-term compliance and benefits from ET.

## Declarations

### Data Availability

No data were generated or analysed for this manuscript.
